# Point-of-Care Ultrasound training in undergraduate education in the European Union: current situation and perspectives

**DOI:** 10.1186/s13089-024-00361-w

**Published:** 2024-02-13

**Authors:** Florence Dupriez, Audrey Hall, Toumane Diop, Alix Collard, Bastian Rodrigues de Castro, Françoise Smets, Andrea Penaloza, Dominique Vanpee

**Affiliations:** 1 Emergency Department, Cliniques Universitaires Saint Luc, Avenue Hippocrate, 10, 1200 Brussels, Belgium; 2 Statistical Support Unit, Cliniques Universitaires Saint-Luc, Brussels, Belgium; 3 Faculty of Medicine and Dental Medicine, Cliniques Universitaires Saint-Luc, UCLouvain, Brussels, Belgium; 4 Institute of Health and Society and CHU UCL Namur, UCLOUVAIN, Brussels, Belgium

**Keywords:** Undergraduate, Medical school, Point-of-care ultrasound, Curriculum

## Abstract

**Background:**

Given the widespread use of Point-of-Care UltraSound (PoCUS) in clinical practice, with ultrasound machines becoming more portable and affordable, recommendations and position statements from ultrasound societies now promote teaching PoCUS in the undergraduate curriculum. Nevertheless, surveys about PoCUS teaching in European medical schools are lacking. This survey aims to overview the current and future undergraduate PoCUS courses in the European Union (EU).

**Results:**

A questionnaire was sent to medical schools in 26 of the 27 countries of the EU; Luxembourg is the only country without a medical school. The survey was completed by the dean or a member of the medical school with knowledge of the medical curriculum. Of the 58 medical schools from 19 countries that responded to the survey, 18 (31.0%) from 13 (68.4%) EU countries reported the existence of an undergraduate PoCUS curriculum and a further 16 (27.6%) from 12 (41.4%) EU countries intended to offer it in the future. No significant difference was observed between the current and future PoCUS curricula regarding its content and purpose. Less than 40 h of theoretical teaching is provided in all the medical schools and less than 40 h of practical training is provided in 12 (75%) of the 16 medical schools which answered this specific question. Of the 40 (69%) surveyed medical schools that do not currently teach PoCUS, 20 (50%) intend to offer PoCUS courses in the future.

**Conclusion:**

Although the lack of teaching hours in curricula suggests that most PoCUS courses are introductory in nature and that medical students are possibly not trained to become autonomous in clinical practice, evaluating the feasibility and impact of PoCUS teaching on clinical practice should be promoted. The medical schools that intend to develop this curriculum should be encouraged to implement validated tools to objectively assess their programs and students’ performances.

## Introduction

The use of PoCUS is becoming increasingly widespread in clinical practice, as ultrasound machines become more portable and less expensive [1]. While several authors have studied the usefulness of PoCUS in clinical practice and promote its usage, others claim a lack of evidence to endorse it [2–5]. The enthusiasm surrounding PoCUS has grown in line with the recommendations and position papers from ultrasound societies and expert consensus statements, which promote and endorse the teaching of PoCUS in medical schools before the postgraduate curriculum [6–9]. A recently published expert consensus recommendation proposed the development of a standardized undergraduate medical curriculum of basic PoCUS training, while advocating for additional research in medical education and PoCUS use in clinical practice [10].

Although surveys about PoCUS teaching have been conducted in North American medical schools and in German-speaking countries using questionnaires sent directly to universities, this has not yet been done at the scale of Europe [11–15]. Some authors previously explored the issue of PoCUS teaching in medical schools in Europe by interviewing the members of organizations promoting PoCUS instead of gathering information directly from the university itself [16]. This survey therefore aims to provide an overview of the current state of PoCUS teaching in the 27 countries of European Union (EU) and to analyze the current and future teaching approaches as well as any barriers to PoCUS training in medical schools.

## Methods

### Study setting

A questionnaire about PoCUS teaching for undergraduate medical students was designed using simple, short, and mostly multiple-choice questions [17]. Questions were designed in a closed-response format and written in English. Respondents could answer in an open-response format if none of the answers seemed appropriate. The questionnaire was constructed using the SurveyMonkey^®^ online questionnaire builder and could not be completed more than once. As the questionnaire did not involve any patients or personal data, ethics committee approval was thus optional. The first part of the questionnaire covered demographic data about the respondent and his/her university, while the second part focused on the availability, description, and content of undergraduate PoCUS courses. If no PoCUS courses were available at the university, respondents were asked about future plans to develop such courses.

### Data collection

Using an internet search, we identified all the universities teaching medicine in the EU as well as the email addresses of their deans and administration offices. We found a total of 285 medical schools in 26 EU countries, with Luxembourg being the only country without a medical school. To reach a confidence level of 95% with a 5% margin of error, a sample size of 164 respondents was required. An email containing an internet link to the survey was sent to the deans of all the identified medical schools. The email was sent in four successive waves between November 9, 2022, and June 15, 2023, to cover the 2022–2023 academic year.

### Statistical analysis

The software SAS 9.4 was used to analyze the anonymized data. Continuous variables describing the study population were expressed using means, standard deviations, and minimum and maximum values. Discrete variables were reported by category as numbers and percentages. Fisher’s exact test was used to compare discrete variables.

## Results

**Fig. 1:**
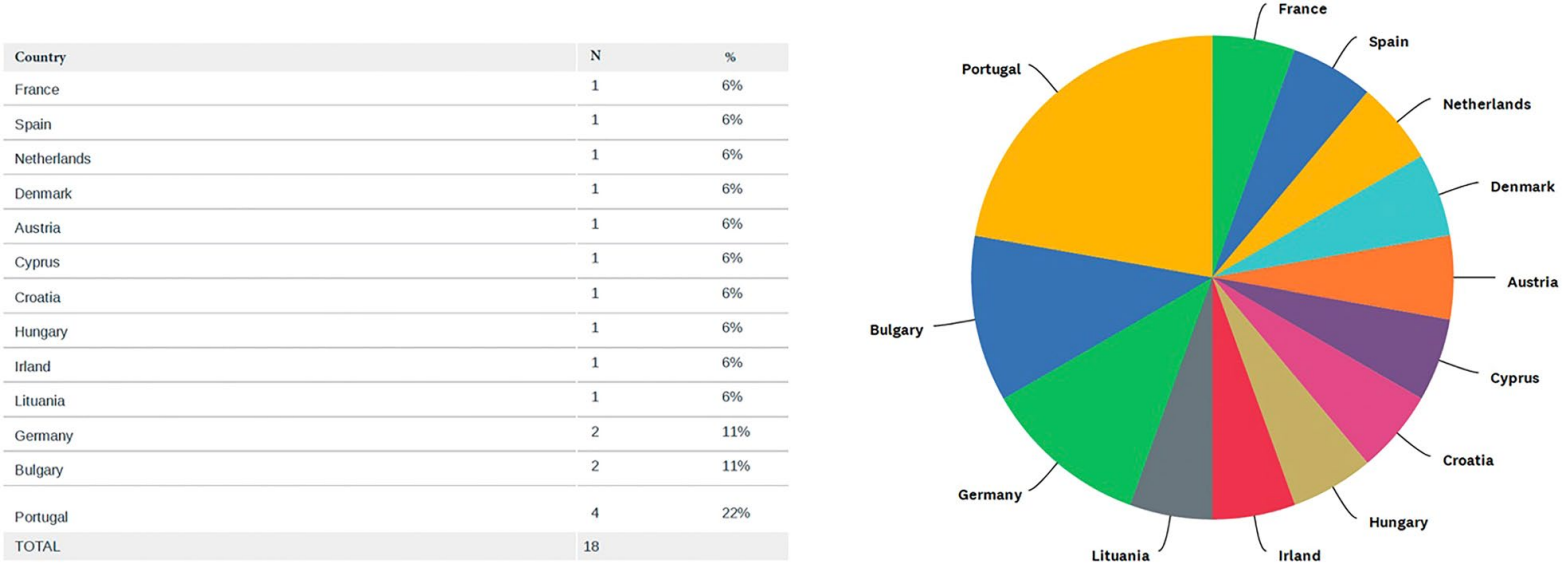
Countries from the European Union with at least one medical school offering a dedicated PoCUS course

**Fig. 2:**
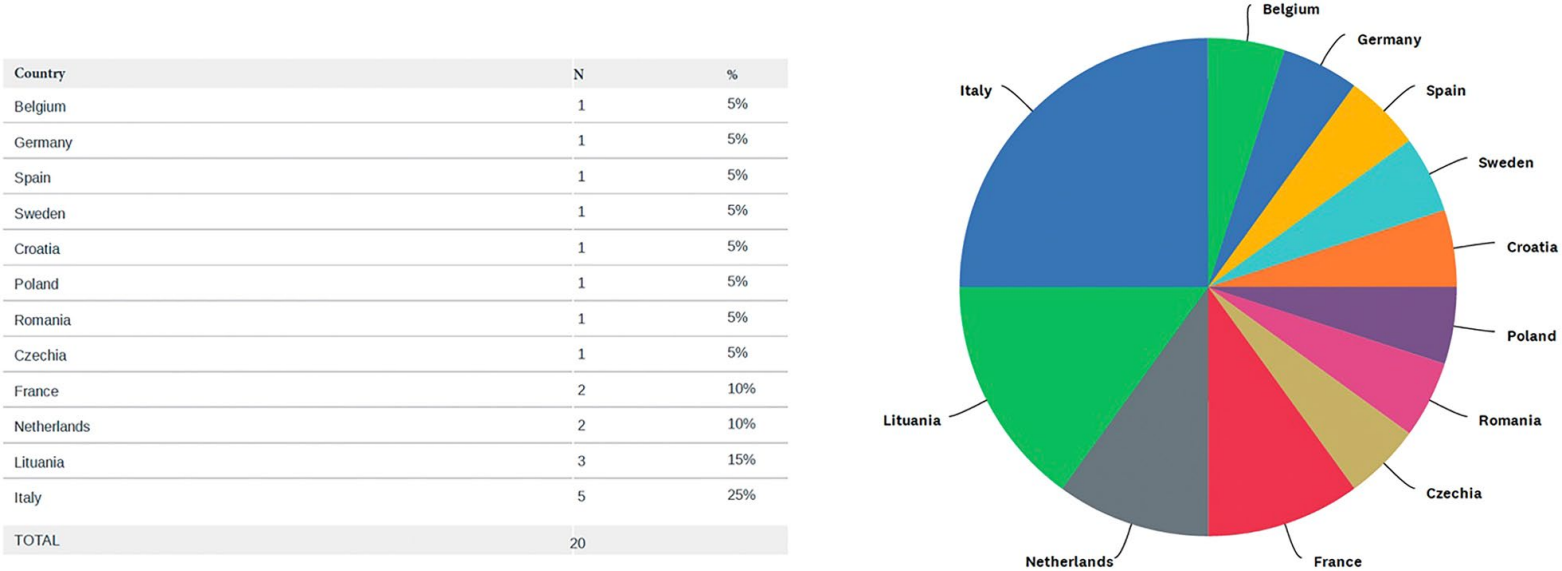
Countries from the European Union with at least one medical school intending to offer a dedicated PoCUS course

Of the 26 EU countries with at least one medical school, 19 (73%) are represented in the survey responses. Figures 1 and 2 illustrate the countries that currently offer or intend to offer a PoCUS course in at least one of their medical schools. Of the 285 medical schools in the EU, 77 (27%) started the questionnaire. Of the 58 (20%) medical schools answering the primary outcome question about whether they had a specific PoCUS course, only 18 (31% [95% CI 20.4–41.7]) currently offer such a curriculum at their institution. The margin of error calculated for 58 respondents is 11.5%. Among the respondents, 38 (65%) provide or intend to provide a dedicated PoCUS course. Table 1 summarizes the profiles of the respondents. A total of 43 (74%) respondents completed the questionnaire, thus allowing secondary outcome analysis. Of the 18 medical schools currently offering a PoCUS course, 16 completed the entire questionnaire. Of these 16 medical schools, 12 (75%) provide theoretical sessions, 15 (94%) practical sessions, and 8 (50%) specific PoCUS training during clinical rotations. The number of students attending the theoretical sessions is more than 100 in nine (56%) institutions, between 50 and 100 in one (6%), and less than 50 in six (38%) institutions. Less than 40 h of theoretical teaching is provided in all the medical schools and less than 10 h in seven of them, while seven (44%) use online teaching. For the practical sessions, the teacher–student ratio is 1 to 4 in three (19%) institutions, 1 to 5 in six (38%), and 1 to more than 5 in seven (44%) institutions. Peer teaching is promoted in nine (56%) medical schools. Less than 40 h of practical training is provided in 12 (75%) medical schools. Table 2 summarizes the contents of the PoCUS courses, the teaching aims, the type of practical sessions, the type of assessments, and the ultrasound machines used. PoCUS is taught from the first to 6th year of medical school and is mandatory in eight (50%) of institutions where it is currently taught. Seven (44%) medical schools report longitudinal teaching of PoCUS over several years, whereas nine (56%) offer transversal teaching in which PoCUS is taught with specific subjects or modules such as anatomy, cardiology, gastroenterology, or pneumology. Assessments are conducted in 12 (75%) medical schools.

**Table 1:** Profiles of respondents

Characteristics	*N* (%) 58 (100)
Function	
Rector, dean, vice-dean	24 (41)
Person aware of or in charge of the undergraduate medical curriculum	31 (53)
Missing value	3
Time in current function	
5 years or less	18 (31)
More than 5 years	39 (67)
Missing value	1

**Table 2:** Comparison of the current and future PoCUS courses

PoCUS teaching	Current *N* (%) (16 respondents)	Future *N* (%) (14 respondents)	*P* value
Subjects taught			
Fundamental principles of ultrasound (physics and knobology)	14 (87.5)	11 (78.6)	0.64^1^
Basic use of ultrasound (probe, mode, settings)	14 (87.5)	12 (85.7)	1.00^1^
Cardiac ultrasound (basics: eyeballing)	10 (62.5)	6 (42.9)	0.46^1^
Cardiac ultrasound (advanced: Doppler and calculation)	5 (31.3)	1 (7.1)	0.18^1^
Lung	8 (50.0)	8 (53.3)	0.73^1^
Procedural ultrasound (central or peripheral catheter, other)	4 (25.0)	7 (50.0)	0.25^1^
Liver/biliary tree	11 (68.8)	8 (57.1)	0.46^1^
Gallbladder	8 (50.0)	7 (50.0)	1.00^1^
Kidney/urinary tract	6 (37.5)	8 (57.1)	0.46^1^
Obstetrical-gynecology (fetal heartbeat, number, position, etc.)	4 (25.0)	2 (14.3)	0.66^1^
Vascular (aorta, deep vein thrombosis)	5 (31.3)	8 (57.1)	0.27^1^
Musculoskeletal	5 (31.3)	4 (28.6)	1.00^1^
Neurology (transcranial Doppler, optic nerve)	2 (12.5)	2 (14.3)	1.00^1^
Thyroid	5 (31.3)	2	0.40^1^
Ultrasound protocols (E-FAST, RUSH, etc.)	12 (75)	13 (93.0)	0.34^1^
Goals			
Improving understanding of physiology	9 (56.3)	8 (57.1)	1.00^1^
Improving understanding of anatomy	9 (56.3)	8 (57.1)	1.00^1^
Improving clinical examination	14 (87.5)	13 (92.9)	1.00^1^
Obtaining appropriate PoCUS images	4 (25.0)	8 (57.1)	0.13^1^
Interpretation of PoCUS images	6 (37.5)	5 (35.7)	1.00^1^
Integration of PoCUS in clinical assessment	8 (50.0)	8 (57.1)	0.73^1^
Improving ultrasound-guided procedures	7 (43.8)	2 (14.2)	0.12^1^
Improving diagnostic accuracy	4 (25.0)	8 (57.1)	0.13^1^
Practical sessions	N (%)	N (%)	
Healthy volunteers	8 (50.0)	10 (71.4)	0.28^1^
Patients	5 (31.3)	7 (50.0)	0.46^1^
Other students	8 (50.0)	6 (42.9)	0.73^1^
Simulators	9 (56.3)	9 (64.3)	0.72^1^
Cadavers	2 (12.5)	1 (7.1)	1.00^1^
Assessment			
Theoretical exam	7 (43.8)	4 (33.3)	0.70^1^
Practical exam on healthy volunteers	7 (43.8)	5 (41.7)	1.00^1^
Practical exam on patients	5 (31.3)	4 (33.3)	1.00^1^
Practical exam on simulators	2 (12.5)	5 (41.7)	0.10^1^
Logbook	2 (12.5)	2 (16.7)	1.00^1^
Other	1 (6.3)	0 (0.0)	1.00^1^
Missing		2	
Specialty of PoCUS educator			
Radiology	6 (37.5)	10 (71.4)	0.08^1^
Internal medicine	10 (62.5)	3 (21.4)	0.08^1^
Surgery	8 (50.0)	4 (28.6)	0.28^1^
Intensive care	8 (50.0)	4 (28.6)	0.28^1^
Emergency physician	10 (62.5)	12 (85,7)	0.01^1^
Anesthesiology	5 (31.3)	4 (28.6)	1.00^1^
Obstetric gynecology	5 (31.3)	4 (28.6)	1.00^1^
Family medicine	6 (37.5)	0 (0.0)	0.02^1^
Ultrasound machines			
Ultraportable	4 (25.0)	3 (21.4)	1.00^1^
Portable	9 (56.3)	9 (64.3)	0.72^1^
Static	8 (50.0)	1 (7.1)	0.02^1^

^1^Fisher’s exact *p* value

Of the 40 (69%) surveyed medical schools that do not currently teach PoCUS, 20 (50%) intend to offer PoCUS courses in the future: three (15%) in the coming year, ten (50%) in the next 5 years, and seven (35%) with an unknown timetable. Only 14 out of 20 respondents intending to offer PoCUS courses in the future continued the questionnaire. In ten (71%) cases, the medical school intended to offer both theoretical and practical courses. PoCUS will be taught from the first to 6th year of medical school and will be mandatory in seven (50%) institutions. Table 2 compares the current and future PoCUS courses.

Of the 20 medical schools that do not intend to teach PoCUS at the undergraduate level, 15 (75%) described a range of different limitations: two (13%) mentioned a lack of financial support, six (40%) reported a lack of ultrasound equipment, six (40%) described a lack of trained instructors, one (7%) was not familiar with PoCUS, and one (7%) cited insufficient evidence in the literature to support PoCUS teaching in the undergraduate curriculum.

## Discussion

Our study shows that the majority of EU countries have at least one medical school in which PoCUS is currently taught or will be taught in the near future. Indeed, the survey includes 19 out of the 26 EU countries with a medical school. Although we were unable to reach the target sample size of 164 respondents, our survey includes the responses of 58 universities compared with previous studies reporting responses from 79 universities in the United States [12], 13 in Canada [14], and 46 in Europe [16]. In their European survey, Prosch et al. reported that 87% of medical schools include PoCUS teaching compared with only 31% in our study. This substantial difference is perhaps due to the selection of universities by PoCUS experts in the study of Prosch et al., which would have induced a selection bias. For this reason, we believe that our results are more representative of the current EU situation, even though the target sample size was not reached. The profile of respondents is also important when determining the accuracy of responses to the sub-questions. Most of the respondents stated that they were the deans or otherwise involved in the undergraduate medical curriculum, which reinforces our conviction that the findings are relevant.

In their 2020 survey, Prosch et al. reported that 40 of the 46 surveyed medical schools included a theoretical ultrasound course and 26 a practical ultrasound course, whereas in our survey, only 12 medical schools reported theoretical PoCUS courses, 15 included practical PoCUS sessions, and eight integrated PoCUS training into clinical rotations. Theoretical, practical, and clinical teaching are the three essential axes for PoCUS learning [18]. Nonetheless, as the methodology of Prosch et al. and our own survey is quite different, an accurate comparison is difficult.

Although we were unable to reach the number of respondents required for a confidence interval of 95% with a 5% margin of error, the number of respondents included in the primary outcome analysis is not trivial, as it reached the mean response rate of online surveys, ranging from 20 to 47% depending on the study [19]. In our survey, very few medical schools currently teach PoCUS, so the results describing the profile of the PoCUS curriculum should be treated with caution. Furthermore, as the aim of the questionnaire was stated in the survey title, this may have favored respondents with a current PoCUS curriculum or intending to develop one in the future. Therefore, we believe that the proportion of medical schools that teach or plan to teach PoCUS is perhaps less than 65% in the EU. Our study did not evaluate the situation beyond the EU and thus did not include the United Kingdom where PoCUS use is widespread. This is another limitation of our findings. The self-administered nature of the survey nevertheless aimed to ensure the privacy and anonymity of responses and avoid interviewer bias.

Many undergraduate PoCUS curricula have been developed and published [20–22], while some have even been prospectively evaluated [23, 24]. Numerous difficulties have nevertheless been reported in integrating PoCUS courses into medical schools. Some studies describe the difficulty in finding qualified teachers, while others are limited by access to ultrasound equipment [25]. Similar concerns were described by our respondents. Indeed, the teacher/student ratio is higher than for other courses, especially for practical sessions and the equipment required is more expensive. Regarding PoCUS teaching, evidence is still required to determine the structure, content, and schedule of the curriculum [5]. Nevertheless, a feasibility study that introduced PoCUS into 1-year anatomy as well as physical examination courses showed that PoCUS courses are well received and perceived as valuable by students [26]. However, students’ appreciation of the curriculum is no guarantee of effective learning. In another study conducted by Liu et al. from 2013 to 2017 on the effect of PoCUS teaching on students’ standardized objective assessments, a statistically significant difference in assessment results for clinical examination performances was found in favor of students undergoing longitudinal PoCUS teaching [27]. In 2018, a scoping review reported how best to integrate PoCUS into the undergraduate curriculum and stressed the possible learning opportunities [28]. For example, informing PoCUS teachers about the body of available literature on the topic of undergraduate PoCUS courses was associated with the best available evidence and future direction for PoCUS teaching in undergraduate education [28]. This review concluded that it was necessary to develop objective tools to assess PoCUS skills and to concentrate efforts on developing PoCUS teaching programs with the intention to deliver robust ultrasound education rather than limiting PoCUS teaching to a few hours [28]. Our survey is consistent with PoCUS teaching in some medical schools, although the number of hours devoted to PoCUS courses is still minimal in the majority of institutions, as the subjects mostly relate to basic ultrasound principles and skills (Table 2). Emphasis is nevertheless given to abdominal PoCUS, as previously reported in a survey of German-speaking medical schools [15]. Less than half of the medical schools that include PoCUS in the curriculum report the use of assessments. The low number of teaching hours combined with the lack of assessments thus suggest that most PoCUS courses are introductory in nature, meaning that medical students are not trained to become autonomous in their clinical practice.

The quality of PoCUS teaching is improved with a multidisciplinary approach [28]. Our survey confirms that PoCUS is taught by a wide range of specialties, including radiology, general medicine, and peer teaching in most medical schools with a dedicated PoCUS course. In comparison to the medical schools currently offering a PoCUS curriculum, the medical schools that intend to introduce it seem to favor instructors with a specialty in emergency medicine. This is perhaps because this specialty is still quite new in some European countries. Indeed, a young specialty may have fewer people involved in university teaching now than in the future. By contrast, the number of instructors trained in family medicine falls drastically (Table 2). The low response rate for course content did not reveal any other significant differences between current and future courses. Nevertheless, the subjects represented are in line with those covered by the expert recommendations [10].

## Conclusion

Our survey shows many countries in the EU have at least one medical school teaching PoCUS or willing to teach PoCUS in the near future. It is essential to define PoCUS education before its widespread dissemination in order to structure and standardize PoCUS education in medical schools in the EU. A recent international consensus conference on PoCUS undergraduate education helped to define the ideal curriculum along with the European Federation of Societies for Ultrasound in Medicine and Biology statement from 2016. It is now crucial to evaluate the feasibility and impact of PoCUS teaching on clinical practice by encouraging the medical schools that intend to develop this curriculum to implement validated tools to objectively assess their programs and students.

## Data Availability

Data are available on reasonable request.

## References

[1] Díaz-Gómez JL, Mayo PH, Koenig SJ (2021). Point-of-care ultrasonography. N Engl J Med.

[2] Hashim A, Tahir MJ, Ullah I, Asghar MS, Siddiqi H, Yousaf Z (2021). The utility of point of care ultrasonography (POCUS). Ann Med Surg.

[3] Casado-López I, Tung-Chen Y, Torres-Arrese M, Luordo-Tedesco D, Mata-Martínez A, Casas-Rojo JM (2022). Usefulness of multi-organ point-of-care ultrasound as a complement to the decision-making process in internal medicine. J Clin Med.

[4] Feilchenfeld Z, Kuper A, Whitehead C (2018). Stethoscope of the 21st century: dominant discourses of ultrasound in medical education. Med Educ.

[5] Feilchenfeld Z, Dornan T, Whitehead C, Kuper A (2017). Ultrasound in undergraduate medical education: a systematic and critical review. Med Educ.

[6] Soucy ZP, Mills LD (2015). American academy of emergency medicine position statement: ultrasound should be integrated into undergraduate medical education curriculum. J Emerg Med.

[7] Cantisani V, Dietrich C, Badea R, Dudea S, Prosch H, Cerezo E (2016). EFSUMB statement on medical student education in ultrasound [long version]. Ultrasound Int Open.

[8] American Institute of Ultrasound in Medicine (2019). Curriculum for fundamentals of ultrasound in clinical practice. J Ultrasound Med.

[9] Dietrich CF, Hoffmann B, Abramowicz J, Badea R, Braden B, Cantisani V (2019). Medical student ultrasound education: a WFUMB position paper. Part I Ultrasound Med Biol.

[10] Hoppmann RA, Mladenovic J, Melniker L, Badea R, Blaivas M, Montorfano M (2022). International consensus conference recommendations on ultrasound education for undergraduate medical students. Ultrasound J.

[11] Bahner DP, Goldman E, Way D, Royall NA, Liu YT (2014). The state of ultrasound education in U.S. medical schools: results of a national survey. Acad Med.

[12] Nicholas E, Ly AA, Prince AM, Klawitter PF, Gaskin K, Prince LA (2021). The current status of ultrasound education in united states medical schools. J Ultrasound Med.

[13] Russell F, Zakeri B, Herbert A, Ferre R, Leiser A, Wallach P (2022). The state of point-of-care ultrasound training in undergraduate medical education: findings from a national survey. Acad Med.

[14] Steinmetz P, Dobrescu O, Oleskevich S, Lewis J (2016). Bedside ultrasound education in Canadian medical schools: a national survey. Can Med Educ J.

[15] Wolf R, Geuthel N, Gnatzy F, Rotzoll D (2019). Undergraduate ultrasound education at German-speaking medical faculties: a survey. GMS J Med Educ.

[16] Prosch H, Radzina M, Dietrich CF, Nielsen MB, Baumann S, Ewertsen C (2020). Ultrasound curricula of student education in Europe: summary of the experience. Ultrasound Int Open.

[17] Passmore C, Dobbie AE, Parchman M, Tysinger J (2002). Guidelines for constructing a survey. Fam Med.

[18] Atkinson P, Bowra J, Lambert M, Lamprecht H, Noble V, Jarman B (2015). International federation for emergency medicine point of care ultrasound curriculum. CJEM.

[19] Nulty DD (2008). The adequacy of response rates to online and paper surveys: what can be done?. Assess Eval High Educ.

[20] Hoppmann RA, Rao VV, Poston MB, Howe DB, Hunt PS, Fowler SD (2011). An integrated ultrasound curriculum (iUSC) for medical students: 4-year experience. Crit Ultrasound J.

[21] Bahner DP, Adkins EJ, Hughes D, Barrie M, Boulger CT, Royall NA (2013). Integrated medical school ultrasound: development of an ultrasound vertical curriculum. Crit Ultrasound J.

[22] Celebi N, Griewatz J, Malek NP, Krieg S, Kuehnl T, Muller R (2019). Development and implementation of a comprehensive ultrasound curriculum for undergraduate medical students—a feasibility study. BMC Med Educ.

[23] Hoppmann RA, Rao VV, Bell F, Poston MB, Howe DB, Riffle S (2015). The evolution of an integrated ultrasound curriculum (iUSC) for medical students: 9-year experience. Crit Ultrasound J.

[24] Heinzow HS, Friederichs H, Lenz P, Schmedt A, Becker JC, Hengst K (2013). Teaching ultrasound in a curricular course according to certified EFSUMB standards during undergraduate medical education: a prospective study. BMC Med Educ.

[25] Shah S, Bellows BA, Adedipe AA, Totten JE, Backlund BH, Sajed D (2015). Perceived barriers in the use of ultrasound in developing countries. Crit Ultrasound J.

[26] Rempell J, Saldana F, DiSalvo D, Kumar N, Stone M, Chan W (2016). Pilot point-of-care ultrasound curriculum at Harvard Medical School: early experience. West J Emerg Med.

[27] Liu RB, Suwondo DN, Donroe JH, Encandela JA, Weisenthal KS, Moore CL (2019). Point-of-care ultrasound: does it affect scores on standardized assessment tests used within the preclinical curriculum? Does point-of-care ultrasound affect scores?. J Ultrasound Med.

[28] Birrane J, Misran H, Creaney M, Shorten G, Nix CM (2018). A scoping review of ultrasound teaching in undergraduate medical education. Med Sci Educ.

